# Genital graft versus host disease in women after allogeneic hematopoietic stem cell transplantation – a single center experience

**DOI:** 10.1007/s00277-025-06224-1

**Published:** 2025-01-28

**Authors:** Yulia Wilk Goldsher, Bina Cohen Sacher, May Cohen, Moshe Yeshurun, Gad Sabah, Ram Eitan, Haim Krissi

**Affiliations:** 1 Department of Obstetrics and Gynecology, The Helen Schneider Hospital for Women, Rabin Medical Center, Petach-Tikva, Israel; 2 Faculty of Medical and Health Sciences, Tel Aviv Univesity, Tel Aviv, Israel; 3 Department of Surgery, University of Toronto, Toronto, Canada; 4 Institute of Hematology, Davidoff Cancer Center, Rabin Medical Center-Beilinson Hospital, Petach Tikva, Israel; 5 Gynecologic Oncology Division, Helen Schneider Hospital for Women, Rabin Medical Center, Petah Tikva, Israel

**Keywords:** Graft versus host disease (GVHD), Genital GVHD, Hematopoietic stem cell transplantation (HSCT), Genitourinary syndrome of menopause (GSM), Vulvar GVHD, Vaginal GVHD

## Abstract

Chronic Graft-versus-host disease (GVHD) is a major complication of allogeneic hematopoietic stem cell transplantation (HSCT), affecting the female genital tract in 25–66% of the patients. This condition, referred to as Genital GVHD is an underdiagnosed gynecologic comorbidity, that can significantly impair quality of life. We aimed to describe the prevalence and management of genital GVHD following HSCT. This retrospective analysis included women who underwent allogeneic HSCT at a single Bone Marrow Transplantation Unit between 2015 and 2020 and were evaluated at a specialized Vulvo-Vaginal Clinic. Diagnosis and severity of genital GVHD were based on the recommendations by the National Institute of Health (NIH), therapeutic options included topical treatments and surgical interventions. Of the thirty-six patients evaluated, 19.4% were diagnosed with genital GVHD. Patients with genital GVHD were older than those with no-genital GVHD (58.42 vs 47.48 years, *p* = 0.02), and most of them had concurrent multi-organ chronic GVHD (85.71%). Genital GVHD was mostly symptomatic in our cohort (71.42%), clinical findings at the time of diagnosis corresponded with NIH grade 3 (severe disease) in 57.1% of cases. Topical treatments were initiated for all patients with genital GVHD, one required surgical intervention. Genitourinary syndrome of menopause (GSM) was diagnosed among 100% of patients with genital GVHD and among 58.62% of patients without genital GVHD (*p* = 0.08). In the genital GVHD group, adherence to clinical follow up was limited (43.85%). Genital GVHD should be considered as part of chronic GVHD evaluation after allogeneic HSCT. It is associated with advanced age and the presence of chronic systemic GVHD. Impaired quality of life and limited follow-up within this population emphasize the need for increased awareness and early evaluations.

**Supplementary Information:**

The online version contains supplementary material available at 10.1007/s00277-025-06224-1.

## Introduction

Women undergoing allogeneic hematopoietic stem cell transplantation (HSCT) are predisposed to multiple gynecological comorbidities, including genital graft-versus-host disease (GVHD), acute-onset menopause with genitourinary syndrome of menopause (GSM), sexual dysfunction, and an increased risk of cervical malignancy [1–15].

Genital GVHD has recently been recognized as a common yet underdiagnosed inflammatory comorbidity, categorized as a manifestation of chronic GVHD. It presents with a wide spectrum of vulvo-vaginal symptoms, including pruritus, provoked or unprovoked vulvodynia, vaginal dryness, and dysuria [16–18]. These symptoms typically occur at least 100 days post-HSCT, most commonly within the first year, though they can appear years later [18]. Chronic inflammation may disrupt vulvar anatomy including; the clitoris, labia and urethral meatus or interfere with vaginal patency. As a result, genito-urinary complications can arise, such as voiding difficulties or provoked vulvo-vaginal pain during sex (dyspareunia) [1, 2, 7, 8, 15, 19–21]. Reported rates of genital GVHD vary widely, ranging from 25 to 66%, likely due to underdiagnosis stemming from low awareness among clinicians, and patients’ reluctance to seek gynecological follow-up [7, 9–13, 17, 18]. Current National Institutes of Health (NIH) guidelines recommend screening all post-HSCT patients for genital GVHD regardless of symptoms and propose clinical severity criteria for diagnosis.[8]

Proposed treatments include topical therapies such as potent steroid ointments, estrogen creams, local immunosuppressive agents [22], or intra vaginal dilators [3, 15, 17, 18]. Surgical interventions are reserved for cases with functional Genito-urinary obstruction, while systemic immunosuppressive treatment is considered for patients with extra-genital chronic GVHD [4, 6, 15, 17, 23]. Current data on risk factors associated with the development of genital GVHD remain limited [4, 20, 24]. This study aims to assess the prevalence and management of genital GVHD in women post-HSCT evaluated at a single designated clinic and to explore baseline characteristics associated with its development.

## Materials and methods

### Study design

In our medical center, all female patients post-HSCT are advised to undergo an evaluation at a specialized gynecological and vulvo-vaginal clinic as part of a routine follow up.

A retrospective search was conducted for all female patients aged ≥ 18 who underwent allogeneic HSCT between January 2015 and December 2020 at the Davidoff Cancer Center, Rabin Medical Center, Israel. We then identified those who attended the recommended evaluations in the designated post-HSCT gynecology clinic and included them in this study. Patients who had at least one clinical assessment were included, patients who passed away during the study period were included until their last follow up. The study was approved by the Rabin Medical Center ethics committee.

Demographic and clinical data were collected retrospectively using electronic medical records. Data included demographics, medical history prior to HSCT (e.g., hematologic disease and related treatments), and post-HSCT events such as diagnosis of acute GVHD, chronic GVHD, associated symptoms, treatments and cytomegalovirus (CMV) reactivation.

Each visit at our designated gynecology clinic included a detailed history and gynecological examination, with documentation of vulvo-vaginal structures (digital and speculum examination), including signs of vulvo-vaginal atrophy, adhesions, narrowing or occlusion of the vaginal opening, urethral meatus occlusion, and other abnormalities.

### Diagnosis of genital GVHD

Diagnosis and severity of genital GVHD were determined based on NIH 2014 guidelines. Menopause was defined as one year of amenorrhea.

### Management of genital GVHD

Patients diagnosed with genital GVHD were offered treatments, including Clobetasol Propionate 0.05% ointment applied twice daily for 14 days, followed by maintenance every three days. Patients with GSM and vulvo-vaginal atrophy were advised to apply Estriol 0.1% cream intravaginally twice daily for 14 days, followed by maintenance every three days. Surgical intervention was offered for cases involving anatomical disruption. Periodic follow-ups were recommended for all patients within the first two years post-HSCT.

### Statistical analysis

Patient demographic and clinical variables were analyzed using Student’s t-test for continuous variables and chi-square tests for categorical variables. Mann–Whitney U tests were used for comparisons as appropriate.

## Results

**Fig. 1:**
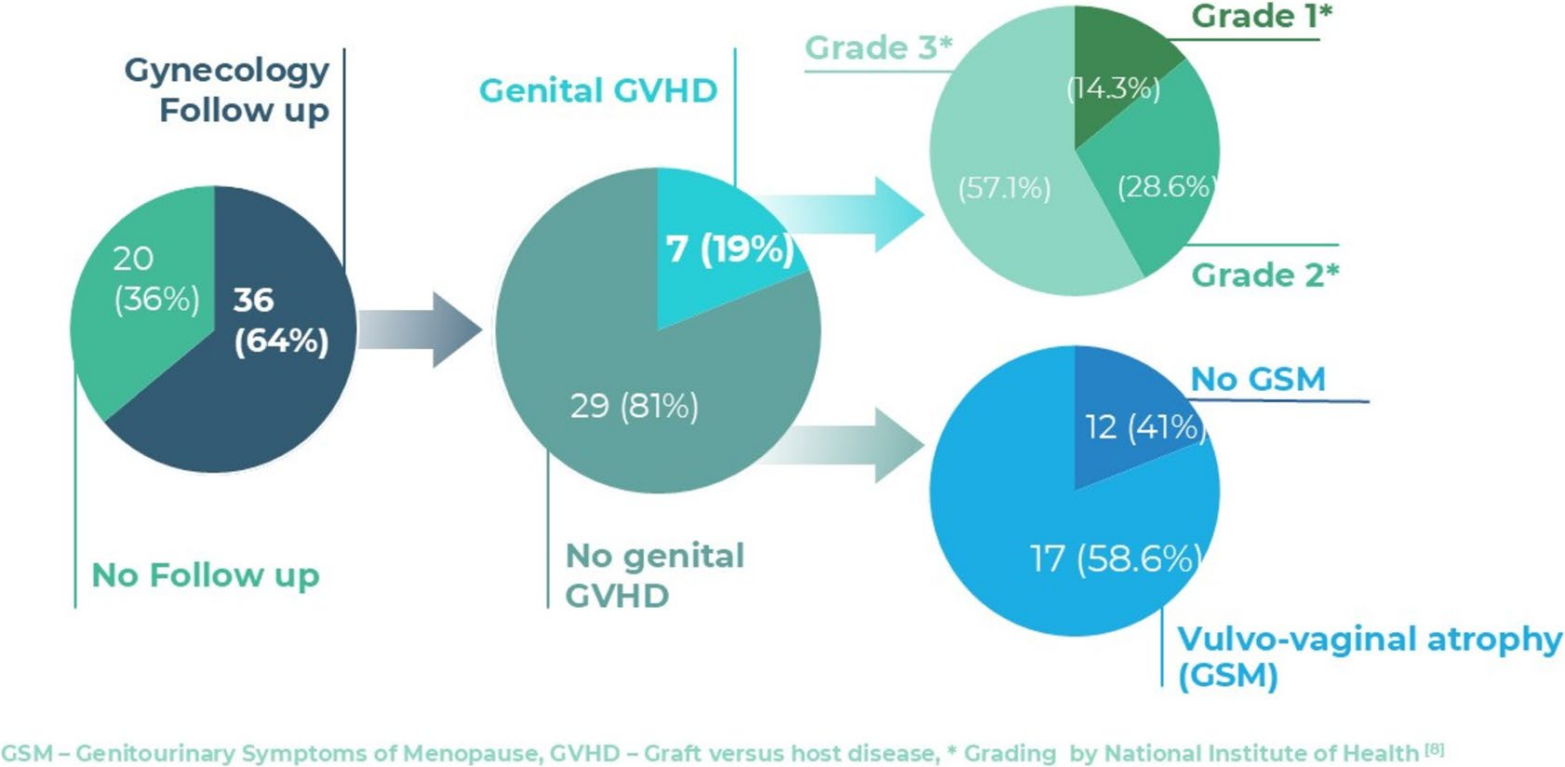
Study cohort, stratified by follow up and diagnosis

Out of 56 women who underwent allogeneic HSCT, 36 (64%) attended an initial evaluation at the designated clinic. Seven patients (19.4%) were subsequently diagnosed with genital GVHD (Fig. 1).

Baseline characteristics are presented in Table 1. Patients with genital GVHD were older than those without (mean age 58.4 ± 8.0 vs. 47.5 ± 16.3 years; *p* = 0.02). Most patients in both groups were postmenopausal and were not sexually active. Acute myeloid leukemia (AML) was the primary indication for HSCT in both groups, with a significantly higher prevalence in the genital GVHD group (85.7% vs. 41.4%; *p* = 0.04). No significant differences were observed in pre-HSCT chemotherapy regimens.

**Table 1:** Patient baseline characteristics stratified by presence or absence of genital GVHD

		Genital GVHD [*n* = 7]	No-genital GVHD [*n* = 29]	P-value
Age, years		58.42 ± 8.4 (48–75)	47.48 ± 16.3 (20–72)	**0.02**
Marital status	Married	6 (85.71%)	17 (58.62%)	0.053
	Divorced/Single/Widow	1 (14.28%)	12 (41.38%)	0.38
Number of children		3.71 ± 1.7 (2–7)	1.93 ± 1.5 (0–6)	**0.03**
BMI		25.75 ± 0.54 (20–38)	22.85 ± 1.23 (15–29)^	0.1
Age of Menarche		13.8 ± 3.54 (12–17)	12.5 ± 1.77 (10–15)	0.7
Current Menopause		7 (100%)	22 (75.86%)	0.48
Sexually active		2 (28.57%)	5 (17.24%)	0.5
Presence of comorbidity*		4 (57.14%)	15 (51.72%)	0.8
Mental health diagnosis**		2 (28.57%)	2 (6.9%)	0.1
Primary hematologic diagnosis	AML	6 (85.71%)	12 (41.37%)	**0.04**
	ALL	1 (14.8%)	3 (10.34%)	0.53
	NHL	0	3 (10.34%)	1
	HL	0	1 (3.44%)	1
	MDS	0	5 (17.24%)	0.56
	MF	0	2 (6.89%)	1
	Other/Unknown	0	3 (10.34%)	1
Chemotherapy treatment before HSCT	Fludarabine	6 (85.71%)	22 (75.86%)	0.57
	Cytoxan	0 (0%)	2 (6.89%)	0.47
	Cytarabine	1 (14.28%)	1 (3.44%)	0.26
	Other/Unknown	0 (0%)	4 (13.79%)	0.57
Immunosuppresion treatment—post HSCT		7 (100%)	19 (65.52%)	0.15
	Cyclosporine	1 (14.28%)	2 (6.89%)	0.48
	Prednisone	1 (14.28%)	5 (17.24%)	1
	Tacrolimus	0 (0%)	1 (3.44%)	1
	Cyclosporine + Prednisone	3 (42.85%)	6 (29.68%)	0.33
	Tacrolimus + Prednisone	2 (28.57%)	4 (13.79%)	0.57
	Lamivudine	0 (0%)	1 (3.44%)	1

Categorical and ordinal Variables are presented as mean ± SD (range) and continuous variables are presented as n (%), SD-Standard Deviation

GVHD—Graft-versus-host disease, BMI – Body mass index, AML-Acute Myeloid Leukemia; ALL- Acute lymphoblastic leukemia, NHL-Non- Hodgkin’s Lymphoma, HL- Hodgkin’s Lymphoma, MDS- Myelodysplastic syndrome, MF- Primary myelofibrosis, HSCT—Hematopoietic stem cell transplantation

^*^ Diabetes Mellitus, Hypertension, Asthma, Allergic rhinitis, Eczema, Urinary tract infection, Temporomandibular Joint Syndrome, Irritable bowel syndrome, Fibromyalgia, Sleep disorder, Thyroid disorder, Systemic Lupus Erythematosus, Oral aphthous, Chronic fatigue

^**^ Anxiety, Depression

^ Data missing for 9 patients

The average time from HSCT to the first gynecological evaluation was extended for the genital GVHD and the no-genital GVHD groups. (mean 23 ± 21 months (range 4–65) vs. 11 ± 10 months (range 3–57); *p* = 0.189).

Table 2 outlines outcomes and complications post HSCT. Acute GVHD occurred in 28.57% of patients with genital GVHD and 34.48% of patients with no-genital GVHD (*p* = 0.77). Extra-genital chronic GVHD was diagnosed in most patients in both groups (100% vs. 79.31%, *p* = 0.19), while multiple organ involvement was more common in the genital GVHD group as opposed to the no-genital group respectively (85.7% vs. 41.4%; *p* = 0.03). To note, none of the patients with genital GVHD experienced CMV reactivation, compared to 51.7% in the no-genital GVHD group (*p* = 0.01).

**Table 2:** Outcomes stratified by presence or absence of genital GVHD

		Genital GVHD [*n* = 7]	No-genital GVHD [*n* = 29]	*P*-value
Acute GVHD		2 (28.57%)	10 (34.48%)	0.77
Diagnosed chronic GVHD		7 (100%)	23 (79.31%)	0.19
Organ involvement	Eyes	7 (100%)	10 (34.48%)	**0.002**
	Gastro-intestinal	6 (85.71%)	17 (58.62%)	0.38
	Lung	3 (42.85%)	2 (6.88%)	**0.004**
	Skin	5 (71.42%)	14 (48.28%)	0.4
Single-organ involvement		1 (14.28%)	11 (37.93%)	**0.04**
Multi-organ involvement		6 (85.71%)	12 (41.37%)	**0.03**
CMV reactivation		0 (0%)	15 (51.72%)	**0.01**
Death during study period		4 (13.79%)	2 (28.57%)	0.34

GVHD—Graft-versus-host disease, CMV – Cytomegalovirus

Vulvo-vaginal symptoms were reported in 71.4% of patients with genital GVHD, with dyspareunia being the most common one (57.1%). Burning sensation (28.57% vs. 3.4%; *p* = 0.03) and unprovoked pain (42.85% vs. 3.4%; *p* = 0.003) were more frequent in the genital GVHD group (Table 3).

**Table 3:** Genital Manifestations and diagnosis stratified by presence or absence of genital GVHD

		Genital GVHD [*n* = 7]	No-genital GVHD [*n* = 29]	*P*-value
Vulvo-vaginal symptoms		5 (71.42%)	14 (48.27%)	0.24
Dyspareunia		4 (57.14%)	10 (34.48%)	0.27
Burning		2 (28.57%)	1 (3.44%)	**0.03**
Unprovoked pain		3 (42.85%)	1 (3.44%)	**0.003**
Itching		1 (14.28%)	2 (6.89%)	0.53
Dryness		1 (14.28%)	6 (29.68%)	0.7
Discomfort		0 (0%)	1 (3.44%)	1
Multiple symptoms		4 (57.14%)	6 (29.68%)	0.09
Grading ^8^	Grade 1	1 (14.28%)		
	Grade 2	2 (28.57%)		
	Grade 3	4 (57.14%)		
Clinical diagnosis				
Vulvo-vaginal atrophy (GSM)		7 (100%)	17 (58.62%)	0.07
Normal		0 (0%)	10 (34.48%)	0.15
Other*		0 (0%)	2 (6.89%)	1

GVHD—Graft-versus-host disease, GSM—Genitourinary Syndrome of Menopause

^*^ Other – clinical findings suggestive of Lichen Sclerosis Atrophicus, Condyloma acuminatum (N = 2)

Among patients diagnosed with genital GVHD, 85.7% were diagnosed during their first gynecological visit, and 57.1% presented with NIH Grade 3 findings (Fig. 1). All patients received topical treatments, and one required surgical intervention for vaginal occlusion. Adherence to follow-up visits was lower among patients with genital GVHD (42.85%), compared to those with no-genital GVHD (75.86%) with a trend towards significance (*p* = 0.089).

## Discussion

This study highlights the prevalence, clinical presentation, and management of genital GVHD among women post-HSCT. The prevalence of 19.4% in our cohort is lower than previously reported rates of 24–66% [3, 7, 18, 25], likely due to underrepresentation of the total HSCT population and low follow-up adherence.

Current guidelines recommend that all patients undergo initial screening for genital GVHD three months post-transplantation [8]. Despite this, the time from transplantation to the first follow-up appointment was much longer for most patients in our cohort which may have contributed to the fact that more than half of the patients with genital GVHD (57.1%) presented with clinical signs and symptoms corresponding to NIH Grade 3, representing long standing inflammation with disruption to vulvo-vaginal anatomy. Delayed gynecological evaluations are likely attributable to multifactorial causes, including limited awareness among both clinicians and patients, as well as the presence of major comorbidities, specifically systemic chronic GVHD, as it has been shown to be highly prevalent in this population with significant disruption to patients' quality of life [10, 11, 14]. Low rate of sexual activity post-HSCT may also contribute to delayed evaluations, as sexual activity can provoke symptoms—particularly pain. Abstinence may provide symptom relief for patients, potentially allowing genital GVHD to progress asymptomatically in some cases, or to postpone gynecological evaluation until symptoms worsen.

Presence of GSM was clinically diagnosed in all patients with genital GVHD, and in more than half of the patients without genital GVHD. While symptoms vary and somewhat differ between patients with GSM only and those with genital GVHD, overlapping complaints are possible. Hence, a low yield of suspicion for genital GVHD should be applied for any vulvo-vaginal symptoms. This may be explained by a baseline low estrogenic environment that contributes to the development of genital GVHD, as demonstrated by the direct association between age and genital GVHD, both in our study and previous data. [8] Given that most patients are in a menopausal state post-HSCT, early evaluations and treatment of GSM are important. Addressing GSM may improve overall quality of life and potentially lower the risk of concurrent genital GVHD development [10, 11, 14].

The association of genital GVHD with age and multi-organ chronic GVHD underscores the need for routine gynecological evaluations in these patients. Notably, genital GVHD can develop as late as 16 years post-HSCT [18], underscoring the value of late evaluations.

Finally, the absence of CMV reactivation in patients with genital GVHD is an intriguing finding that warrants further investigation.

Low adherence to follow-up in our cohort as well as in previous studies [2, 18] remains a critical barrier to improving outcomes and requires increased awareness among clinicians and patients. In addition, the high prevalence of sexual abstinence among the cohort emphasizes the importance of direct questioning, aiming for early diagnosis and treatment of both genital GVHD and GSM, which may aid in patients' sexual rehabilitation.

The retrospective nature and small sample size of this cohort study may limit the generalizability of the findings and the statistical power, potentially influencing the ability to detect possible associations. In addition, the retrospective design lacks some data variables that could be relevant; like HSCT protocols, diagnosis and treatments received at other medical centers and patient-reported outcomes. The lack of consistent follow-up data for a significant portion of the cohort further limits the ability to evaluate long-term outcomes and treatment efficacy.

Nonetheless, this study provides valuable insights into clinical features and treatment of genital GVHD. Early evaluation and treatment of GSM and genital GVHD may improve quality of life and potentially reduce disease progression.

## Conclusion

Genital GVHD is a significant yet underdiagnosed condition in women post-allogeneic HSCT. It is associated with advanced age and multi-organ chronic GVHD. Augmented awareness, early evaluation as part of chronic GVHD screening and treatment can advance understanding, improve follow-up care, and enhance outcomes and well-being in this population.

## Supplementary Information

Below is the link to the electronic supplementary material.

Supplementary file1 (JPG 111 KB)

## Data Availability

The data supporting the findings of this study are not publicly available due to their sensitive nature and the risk of identifying participants. The small sample size, coupled with the specificity of demographic and clinical characteristics, increases the likelihood of participant re-identification. Additionally, the inclusion of rare medical conditions and the potential for data linkage with publicly available datasets further amplify these risks. Sharing these data would compromise confidentiality, as the study was conducted in Israel, in compliance with ethical guidelines set forth by the Helsinki Committee and the Protection of Privacy Law (1981). This decision also aligns with international ethical standards, including the Declaration of Helsinki. For further inquiries, researchers may contact the corresponding author, subject to ethical and legal considerations.
