# Supplementary figures and images for: Genital graft versus host disease in women after allogeneic hematopoietic stem cell transplantation – a single center experience
Source: Ann Hematol. 2025 Jan 28;104(1):773–9. doi: 10.1007/s00277-025-06224-1 (PMC11868347; doi:10.1007/s00277-025-06224-1)

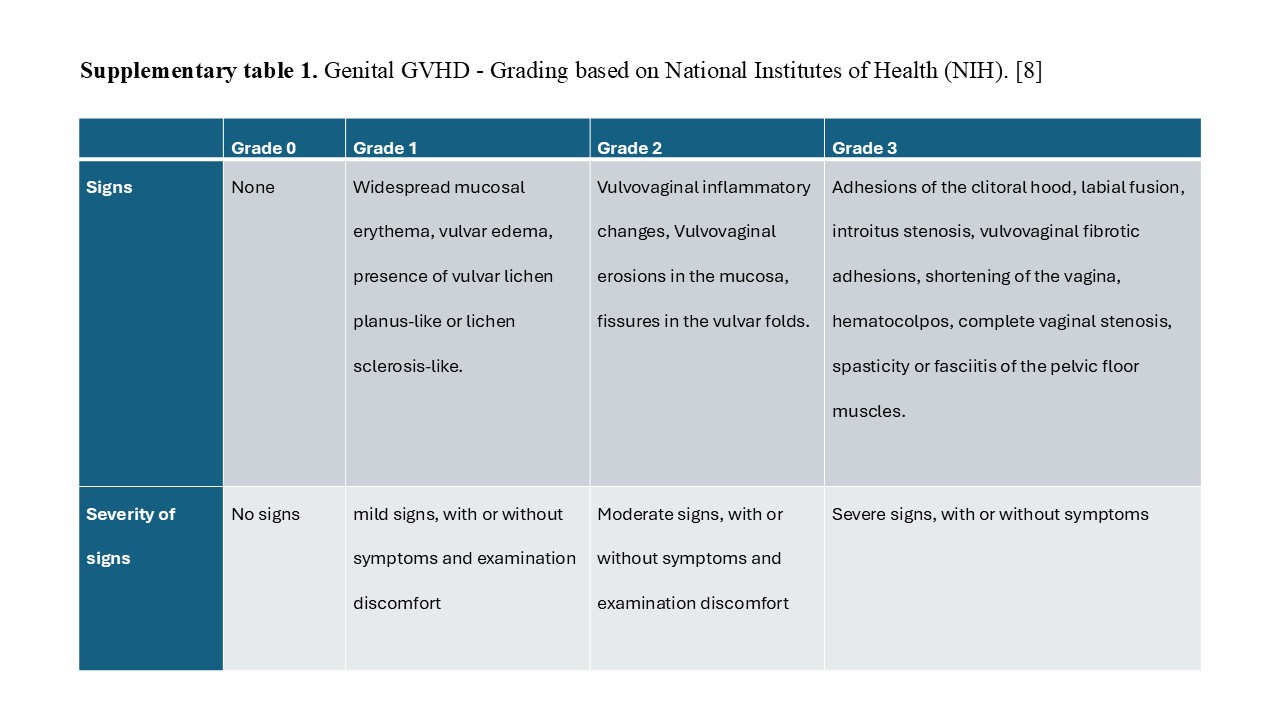

Supplement: Supplementary file 1 — Supplementary file1 (JPG 111 KB) [file 277_2025_6224_MOESM1_ESM.jpg]
